# Estimating the photo-fission yield of the *Trinity Test*

**DOI:** 10.1038/s41598-020-61201-0

**Published:** 2020-03-06

**Authors:** F. A. Khan

**Affiliations:** Department of Physics, Govt. Degree College Pulwama, Higher Education Department, Srinagar, 190009 J & K India

**Keywords:** Energy science and technology, Physics

## Abstract

In the *Trinity Test*, the energy was released not only from the fission of Pu-239 core but also from the fast neutron fission of tamper surrounding it. The high energy photons produced from the fissioning core can also induce fission in U-238 tamper. In this work, an effort has been made, for the first time, to develop an approach to investigate the possibility of photon-induced fission in U-238 tamper used in *Trinity Test* nuclear weapon. GEF nuclear reaction code, version 2018/1.1 is used to calculate the prompt γ-spectrum from the fast fission of Pu-239 core of the device. It is estimated that 14.3 ton (t) of TNT of the total 21 kT yield of the *Trinity* nuclear device is contributed by the photo-fission of U-238 tamper.

## Introduction

When the first nuclear device, called the *Gadget*, was tested in 1945, there were several comments regarding the amount of energy released in the test. The device worked on the fission induced by fast neutrons (*E* ~ MeV) and so do all the modern nuclear devices. Its design, except some minor changes, was the same as that of the one used on Nagasaki - an implosion-type plutonium based device^1^, using 6.19 kg of highly enriched plutonium^2^.

Only when the photographs of the *Trinity Test* fireball, taken by Julian Mack and his team, were declassified, the British physicist Sir G. I. Taylor studied the problem of nuclear explosion in detail and obtained the expression for the energy released. He gave two values of yield as 16.8 kT and 23.7 kT^3,4^. The energy released from the *Trinity Test* nuclear device was not only from the neutron-induced fission of Pu-239, but also from the fission of U-238 tamper by fast neutrons (*E* ~ MeV) released from the Pu-239 fissioning core^5^. A tamper is a heavy material surrounding the fissile material core, that not only reduces its critical mass, when added sufficiently, by reflecting neutrons back to the fissioning core, but also retards the inevitable expansion of the core, allowing more time for fissions to occur until the core density drops to the value where criticality no longer holds. In *Gadget*, the plutonium core, besides highly explosive chemicals, was wrapped by about 120 kg natural uranium tamper^5^. In addition to other forms of energy, the fissioning core also releases fast neutrons and prompt γs. So, keeping in view the formation of γs, it becomes interesting to study whether there is any contribution in the energy released from the γ-induced fission of U-238 tamper. While looking for the literature relevant to the γ-induced fission of U-238 etc. tamper in a nuclear device, I have not come across any research article.

In the present work, an approach is developed to investigate the possibility of photon-induced fission of U-238 tamper generated by the fission of Pu-239 core of the *Trinity* nuclear device. U-238 nuclei can undergo fission by such photons, particularly in the Giant Dipole Resonance (GDR) energy region^6^. Here, GEneral Fission (GEF) nuclear reaction code^7–9^ is used to calculate the prompt γ-spectrum from the fissioning Pu-239 core of the device. For the first time, the effort of investigating the photo-fission of nuclear device tamper is being made.

## Calculation and Results

The assumptions made in the present work and upon which the present calculation is based are stated below:

1. The geometry of the initial nuclear fireball produced in the *Trinity Test* is spherical. This seems to be justified as our calculation deals with the very initial stage of the explosion.
2. The γs generated from the fission of tamper itself are not considered in calculating the yield.
3. The absorption of γs by the Pu-239 core is not considered.

The yield of the *Trinity Test* is taken as 21 kT from^10^. As the yield of the *Trinity* nuclear device has a 30.8% (i.e., 6.5 kT of 21 kT) contribution from the U-238 tamper fast-neutron fission^5^, so the mass that gave 21 kT yield also includes some quantity of the tamper. Thus the 14.5 kT energy released from the Pu-239 fissile core has to fission mass

1 $${M}_{fis}=\frac{Yield\,({\rm{MeV}})\times {m}_{1}}{207\,({\rm{MeV}})}$$

where *m*_1_ is the mass of Pu-239 nucleus and energy released per Pu-239 nucleus fission is taken as 207 MeV.

2 $${\rm{Or}},{M}_{fis}=726\,{\rm{g}}$$

Using mass-energy equivalence relation, 14.5 kT yield has to consume 0.67 g of Pu-239 core.

Now, the time required to fission 726 g of Pu-239 mass, initiated by neutrons, is calculated from the number of generations *G* and the time per generation *τ* from^11^ as

3 $${t}_{fis}=\tau G=\tau \frac{\mathrm{ln}(N)}{\mathrm{ln}(\nu )}$$

where $\tau =\frac{{\lambda }_{fis}}{{v}_{n}}$. *λ*_*fis*_ is the fission mean free path for neutrons in Pu-239 core, *v*_*n*_ the neutron velocity. *N* is the number of nuclei to be fissioned, *ν* the average number of neutrons released per fission. While, in *λ*_*fis*_ = 1/*nσ*_*fis*_, *σ*_*fis*_ is the fission cross section and *n* the Pu-239 nuclei number density. Using the mass of one Pu-239 atom and above value of fissioned Pu-239 mass, *N* comes out to be 1.8×10^24^. For Pu-239, *ν* is taken as 3.16 for 2 MeV neutron energy from^12^, and only half of the neutrons produced are treated as effective in causing the fission of Pu-239 core. The velocity of neutrons corresponding to 2 MeV energy is 1.96 × 10^7^ m/s. Besides, *σ*_*fis*_ at 2 MeV neutron energy is taken as 1.97 b from ENDF/B-VIII.0 Library^13^. The time to fission using Eq. (3) and other calculated values of different quantities are given in Table 1.

**Table 1:** Values of different quantities related to calculating the time to fission Pu-239 core nuclei.

Quantity	Value
*M*_*fis*_ (g)	726
*M*_*cons*_ (g)	0.67
*N*	1.8×10^24^
*τ* (s)	6.58 × 10^−9^
*λ*_*fis*_ (cm)	12.9
*t*_*fis*_ (μs)	0.7

### Calculation with GEF code

GEF is a Monte-Carlo code which combines general laws of quantum and statistical mechanics with specific experimental information developed to model low energy nuclear fission process^7–9^. It treats spontaneous fission and induced-fission up to an excitation energy of about 100 MeV of a wide range of heavy nuclei ranging from polonium to seaborgium. GEF calculates pre-neutron and post-neutron fission-fragment nuclide yields, angular momentum distributions, isomeric yields, prompt-neutron yields and prompt-neutron spectra, prompt-γ spectra, and several other quantities for the fissioning nuclei.

As no experimental data of prompt-gamma multiplicity distribution is available for fast neutron fission (*E*_*n*_ = 4.3 MeV here, see below) of Pu-239, so to calculate the number of photons in our concerned energy range, GEF nuclear reaction code, version 2018/1.1^7^ was used to calculate the prompt γ-spectrum from the neutron-induced fission of Pu-239 core. In the Manhattan Project, neutron sources utilized (α, *n*) reaction, where an α-emitting element is mixed with a low *Z* material, usually Be-9^14,15^. In such a reaction, the average energy of the neutron emitted is 4.3 MeV. This average value was taken as the incident neutron energy that initiated the chain reaction in Pu-239 core, and at which the prompt γ-spectrum was calculated from GEF code. A total of 10^5^ events were simulated at 4.3 MeV incident neutron energy. GEF provides the prompt photon count as a function of photon energy interval (bins). The energy values mark the lower limit of the corresponding energy bins. GEF prompt γ-spectrum has been taken from 5 MeV and onwards (upto 15.7 MeV, the energy value GEF provides for prompt-γs at 4.3 MeV incident neutron energy). The reason for choosing 5 MeV as the lower limit of the energy interval is that the photo-fission cross section for U-238 starts taking significant values from this energy, and the photons having energy less than 5 MeV are not capable of causing the U-238 fission.

**Figure 1:**
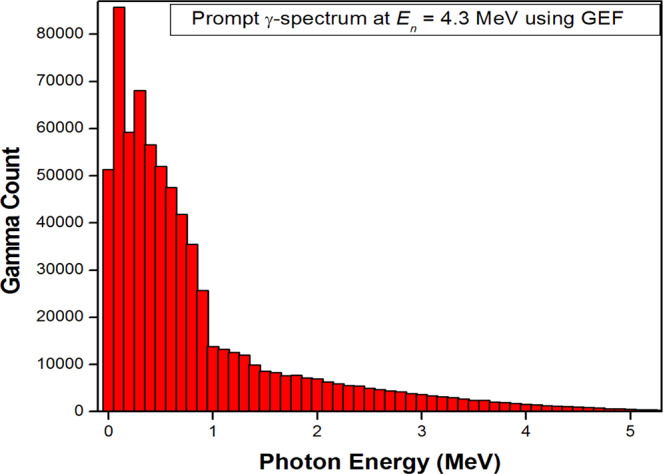
Histogram showing the γ count versus γ-energy for *n*-induced fission of U-238 at *E*_*n*_ = 4.3 MeV, using GEF nuclear reaction code.

Figure 1 shows the histogram obtained for prompt γ-spectrum from neutron-induced fission of Pu-239. The photon count from the fissioning Pu-239 core has been plotted as a function of energy bins to have a look at the number of photons capable of causing U-238 fission. From the figure, it is seen that the production of γs decreases sharply with increasing photon energy, shown by the skewed right histogram. At lower energies, the photon count reaches of the order of 10^5^, while as it reduces to about 1 at the middle energies of the energy interval. Though the mean γ-multiplicity, ${\bar{M}}_{\gamma }$ obtained here is 7.167, but this value represents the data from 0 – 15.8 MeV; hence this mean value willn’t represent our data (photon energy capable of causing U-238 fission) which encompasses higher energy values and whose count is very very small.

To give some idea of the number of fissions in the tamper by photons concerned, mean energy per photon, ${\bar{E}}_{\gamma }$ was calculated for the histogram over the energy interval 5–15.8 MeV by the ratio of the total photon energy to the total photon count. The value comes out to be 5.6 MeV. Now to calculate the ${\bar{M}}_{\gamma }$, using relation ${\bar{E}}_{{\rm{Tot}}}={\bar{M}}_{\gamma }\times {\bar{E}}_{\gamma }$, total mean γ energy per fission ${\bar{E}}_{{\rm{Tot}}}$ is taken from GEF code (6.537 MeV here). ${\bar{M}}_{\gamma }$ value reduces to 1.17 per fission.

Taking the mass of the highly enriched Pu-239 spherical core of the *Trinity* nuclear test to be 6.19 kg^2,11^, and as calculated above taking 726 g of this mass that fissioned, the number of Pu-239 nuclei in 726 g comes out to be 1.8 × 10^24^. Taking the ${\bar{M}}_{\gamma }$ as 1.17, the number of γs emitted by 726 g Pu-239 core comes out to be 2.1 × 10^24^.

In implosion-type nuclear weapons (like the *Trinity* one), the surrounding explosive materials are made to crush the fissile core to critical density, and then neutrons initiate the nuclear chain reaction. γs emitted by the fissile core get absorbed by the surrounding material. So, the number of γs causing the fission of U-238 nuclei are calculated by using the formula

4 $${N}_{react}={N}_{ini}\{1-\exp (-\frac{3}{4}{\sigma }_{{\rm{fis}}}^{{\rm{U}}}{n}_{U}R)\}$$

where *N*_*ini*_ is the initial number of γs, which is 2.1 × 10^24^ as calculated above. ${\sigma }_{{\rm{fis}}}^{{\rm{U}}}$ is the fission cross section of γs for U-238 at ${\bar{E}}_{\gamma }$= 5.6 MeV and *n*_*U*_ is the U-238 nuclei number density (4.77×10^28^/m^3^). *R* is the radius of the sphere. Equation (4) is valid for a linear distance, while as the *Trinity* nuclear device had the spherical interior structure (core and tamper), so the γs are assumed to be emitted in random three-dimensional directions. So, the linear distance *R* was replaced by the average distance from any point within a sphere to its surface (= $\frac{3}{4}R$ of the sphere as given by Calculus).

**Figure 2:**
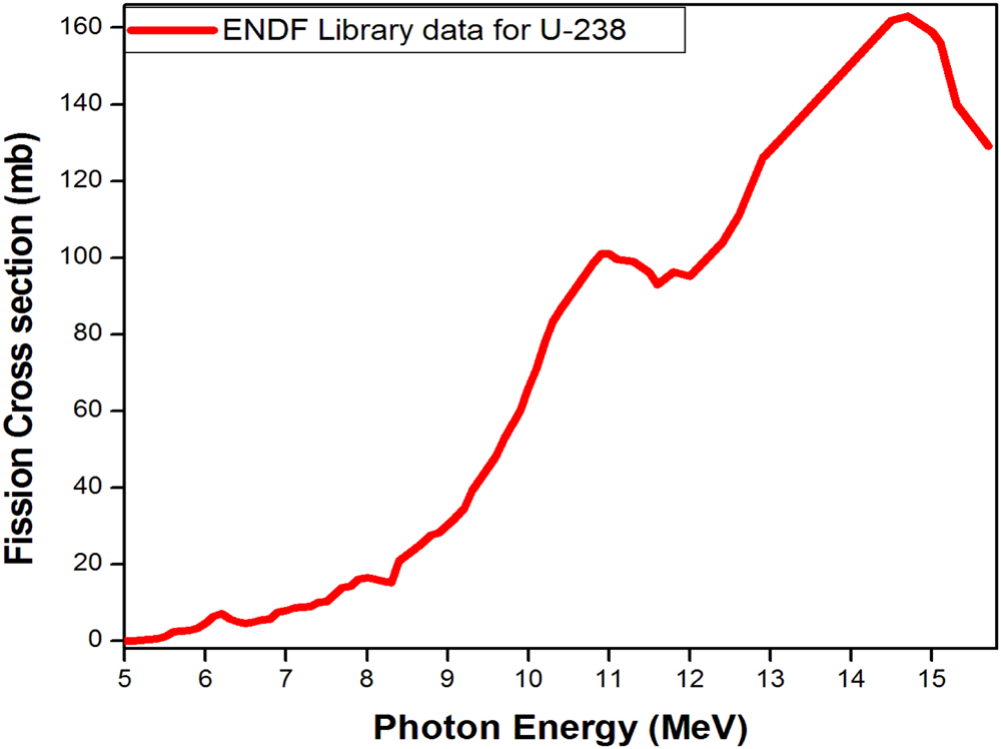
Photo-fission cross section for U-238 versus photon energy from ENDF Library.

As the *Trinity* nuclear device starts expanding soon after the initiation of the nuclear chain reaction, all the quantities in the above equation change. ${\sigma }_{{\rm{fis}}}^{{\rm{U}}}$ varies with the γ energy as shown in Fig. 2 based on ENDF Library data for U-238 photo-fission^16^. For our calculation, ${\sigma }_{{\rm{fis}}}^{{\rm{U}}}$ = 0.0024 b was taken for the mean γ energy ${\bar{E}}_{\gamma }$= 5.6 MeV as calculated above for γ energy interval 5–15.8 MeV.

*n*_*U*_ and *R* values also vary with the expansion but as our calculation deals with first few micro-seconds of the explosion, so their values are taken as constant. Next, as the *Gadget* and *Fat Man* were fundamentally the same in design except some minor changes, so the value of *R* (outside diameter of the U-238 tamper shell) is taken as 0.1143 m^17^.

Using all the above values, Eq. (4) shows that less than 1% of the initial number of γs survives for fission and the rest escape. This fission causing γ number reduces to 2.1×10^21^. Taking the energy released per fission as 180 MeV, the amount of energy released due to photo-fission comes out to be 6 × 10^10^ J. This amounts to 14.3 ton of TNT of the total 21 kT yield of Trinity nuclear device. The number will be less when the absorption of γs by the fissioning fissile core is taken into account.

Now, 30.8% of the total yield of the *Trinity* nuclear test from the fission of uranium tamper corresponds to 6.5 kT of energy. Hence as per our calculation, the energy released from the photo-fission of U-238 tamper is just 0.2% of the total energy released from the fission of uranium tamper of the *Trinity* nuclear device. Taking the abundance of U-235 as 0.72%, 120 kg natural uranium tamper used in the *Gadget*^5^ will contain 0.864 kg of U-235. As U-235 nuclei concentration in the tamper is very low, so its contribution has not been considered in the present case.

## Conclusion

In the *Trinity Test* nuclear explosion, the energy released has been found to have a small contribution from the photo-fission of U-238 tamper, and the estimate of the contribution has been given for the first time.

Less than 1% of the γs produced by the fissioning Pu-239 core survives to cause U-238 tamper fission. 14.3 ton of TNT energy is estimated to be released from the photo-fission of the tamper. This energy is less than 1% of the total energy released from the fission of U-238 tamper of the device.
