# Author Correction: Estimating the photo-fission yield of the *Trinity Test*

**DOI:** 10.1038/s41598-021-95943-2

**Published:** 2021-08-06

**Authors:** F. A. Khan

**Affiliations:** Department of Physics, Govt. Degree College Pulwama, Higher Education Department, Srinagar, J & K 190009 India

Correction to: *Scientific Reports* 10.1038/s41598-020-61201-0, published online 06 March 2020

The original version of this Article contained a repeated error, where the value of ton (of TNT) unit of energy “14.3” was incorrectly given as “14.5”.

As a result, in the Abstract,

“It is estimated that 14.5 ton (t) of TNT of the total 21 kT yield of the *Trinity* nuclear device is contributed by the photo-fission of U-238 tamper.”

now reads:

“It is estimated that 14.3 ton (t) of TNT of the total 21 kT yield of the *Trinity* nuclear device is contributed by the photo-fission of U-238 tamper.”

In the Calculations and Results section, under the subheading ‘Calculation with GEF code’,

“This amounts to 14.5 ton of TNT of the total 21 kT yield of Trinity nuclear device.”

now reads:

“This amounts to 14.3 ton of TNT of the total 21 kT yield of Trinity nuclear device.”

In the Conclusion,

“14.5 ton of TNT energy is estimated to be released from the photo-fission of the tamper.”

now reads:

“14.3 ton of TNT energy is estimated to be released from the photo-fission of the tamper.”

The original Article has been corrected.

